# P01.54. Electrical potential of acupuncture points: use of a Scanning Kelvin Probe

**DOI:** 10.1186/1472-6882-12-S1-P54

**Published:** 2012-06-12

**Authors:** A Ahn, B Gow, S Smith, I Baikie

**Affiliations:** 1 Massachusetts General Hospital Martinos Center for Biomedical Imaging, Charlestown, USA; 2 Kelvin Probe Technology, Wick, United Kingdom

## Purpose

According to conventional wisdom within the acupuncture community, acupuncture points are distinguishable by their electrical properties. However, confounders arising from skin-to-electrode contact (such as electrode pressure and skin moisture) have contributed to controversies over this claim. Because the Scanning Kelvin Probe relies on capacitive coupling and thus measures electrical potential without actually touching the skin, it is ideal for assessing the electrical characteristics of acupuncture points. In this study, we evaluate the electrical potential profiles of acupoint LI-4 and PC-6 and their adjacent controls. We hypothesize that acupoints are associated with a potential maximum, while adjacent controls are not.

## Methods

Three healthy individuals were recruited for this study (towards an anticipated total of 24). The right arm was immobilized and positioned for measurements. Acupuncture points LI-4 and PC-6 and their adjacent controls (1.0 cm radial) were investigated. A 2-mm probe tip was placed over the predetermined skin site and adjusted to a tip-to-sample distance of 100 µm under tip oscillation settings of 94.8Hz frequency. A 6x6 surface potential scan spanning a 9mm x 9mm area were obtained.

## Results

In two of three subjects, a potential maxima of greater than 60 mV above the mean of the scanned area was seen at LI-4. This maxima was approximately 2mm in diameter and reproducibly seen with separate consecutive scans. No such maxima was seen in its adjacent control. No identifiable maxima was seen with either PC6 or its control.

## Conclusion

Preliminary data suggests that acupoint LI-4 is associated with a 60 mV peak in surface potential while no such peak was identified at PC-6. Additional data will be collected in additional subjects to assess the generalizability of this finding.

